# Comparative Analysis of 24-h Movement Behaviours in Non-Overweight and Overweight/Obese Children: Findings from the FAMIly Physical Activity, Sedentary Behaviour, and Sleep (FAMIPASS)

**DOI:** 10.3390/children11111298

**Published:** 2024-10-27

**Authors:** Erik Sigmund, Jaroslava Voráčová, Jan Dygrýn, Michal Vorlíček, Dagmar Sigmundová

**Affiliations:** 1 Institute of Active Lifestyle, Faculty of Physical Culture, Palacký University Olomouc, 77900 Olomouc, Czech Republic; erik.sigmund@upol.cz (E.S.); jan.dygryn@upol.cz (J.D.); michal.vorlicek@upol.cz (M.V.); 2 Department of Social Sciences in Kinanthropology, Faculty of Physical Culture, Palacký University Olomouc, 77900 Olomouc, Czech Republic; jaroslava.voracova@upol.cz

**Keywords:** physical activity, sedentary, excessive body weight, gender, socioeconomic status

## Abstract

Background: Childhood overweight and obesity are global health concerns associated with insufficient physical activity (PA), excessive sedentary behaviour (SB), and inadequate sleep. This study aimed to determine whether differences exist in 24 h movement behaviours between Czech non-overweight children and children with overweight/obesity aged 3–10 years, with respect to their gender, age, or family socioeconomic status (SES). Methods: A total of 381 children (49.9% girls), aged 3–10 years, participated. Their PA, SB, and sleep were continuously monitored over a regular week using wrist-worn accelerometers. Weight status was determined by BMI z-scores, according to World Health Organization standards. SES was assessed using the Family Affluence Scale. Results: Non-overweight children had averages of 414 min of PA, 472 min of SB, and 554 min of sleep per day, while children with overweight/obesity had averages of 392 min of PA, 503 min of SB, and 545 min of sleep. Non-overweight children engaged in significantly more PA (22 min per day; *p* = 0.014) and significantly less SB (31 min per day; *p* = 0.002) than children with overweight/obesity. No significant differences were found between the weight groups in gender distribution, age, family SES, or maternal and paternal obesity. Conclusions: Non-overweight children exhibited higher levels of PA and lower levels of SB compared to their counterparts with overweight/obesity, independent of gender, age, or family SES. These findings highlight an association between daily movement behaviours and weight status in young children. Further research is needed to explore the impact of modifying PA and SB on weight outcomes.

## 1. Introduction

Childhood overweight and obesity have become significant public health concerns globally, affecting children and adolescents across low- to high-income countries. In the last two decades, there has been a notable increase in excess body weight among preschool children in countries, such as Poland [1], Germany [2], Sweden [3], various Southern European nations [4], and even in less economically developed countries [5]. The World Health Organization (WHO) has highlighted the preschool years as a critical period for the development of excess body weight and related metabolic disorders, with most preschool children who are overweight or obese continuing to be above a healthy weight as they grow older [6]. These trends emphasize the urgency of initiating prevention and treatment efforts before children reach 5–7 years of age [7].

Optimal movement behaviours within a 24 h period—comprising physical activity (PA), sedentary behaviour (SB), and sleep—are integral to children’s health and development. Favourable associations between adherence to 24 h movement behaviour recommendations—sufficient daily moderate to vigorous physical activity (MVPA), limited SB, and adequate sleep duration and positive health outcomes, such as reduced adiposity, improved fitness, or better cardiometabolic health—have been repeatedly demonstrated in school-aged children, adolescents, and adults [8,9]. However, the relationship between compliance with these guidelines and body composition has not been clearly established in preschool children [8,9]. This gap may be due to methodological challenges, including the cut-off approach for assessing movement behaviours and the intermittent, variable intensity of PA in preschoolers [10].

Among school-aged children and adolescents, family socioeconomic status (SES) and gender have been consistently identified as determinants of movement behaviours [11,12,13], which significantly contribute to the prevalence of obesity, disproportionately affecting certain genders and SES groups [14,15]. In European preschool children aged 2–7 years, higher prevalence of excess body weight has been observed in girls compared to boys in most countries, except for Czechia, Germany, and Serbia [1,4]. Additionally, children from families with lower SES are more likely to be overweight or obese [1,2,3]. Significant correlates of excessive body weight in this age group include parental obesity, lower maternal and paternal education levels, excessive screen time [1], and consumption of sweetened beverages [4].

Despite the recognized importance of early childhood in obesity prevention, research focusing on the relationship between 24 h movement behaviours and body weight in preschool children remains limited, particularly in Central European countries. The countries of Central Europe, especially those from the post-communist bloc, are underrepresented in systematic reviews and meta-analyses examining movement behaviours and adiposity. Czechia, for example, has been sporadically included in such studies [16,17], with only one study in the past two decades focusing on children and adolescents aged 8–18 years [17]. This lack of data hinders the development of targeted interventions in these regions.

To address this gap, the current study aimed to explore the relationship between 24 h movement behaviours and body weight levels in Czech children under 10 years of age. By focusing on the similarities and differences in movement behaviour patterns between non-overweight children and those with overweight/obesity, this research sought to provide insights that could be used to inform the design of effective interventions tailored to this population. Thus, the main objective of this study was to determine whether differences exist in 24 h movement behaviours between Czech non-overweight children and children with overweight/obesity aged 3–10 years, with respect to gender, age, and family SES.

## 2. Materials and Methods

### 2.1. FAMIPASS Study

FAMIPASS is a nationally representative cross-sectional study focused on the analysis of 24 h movement behaviour of families with young children [18]. In this study, a family is defined as one or more parents and their children living together as a unit. Participating families were approached through preschools and primary schools from a stratified sample of urban and rural areas in Bohemia, Moravia and Silesia. Children and their parents were measured in their usual daily routine and environment. We planned to measure a total of 500 families [18]. The sample size was determined based on a statistical significance level of α = 0.05, an effect size of 0.5, and a statistical power of 0.8, taking into account an expected 20% mortality in the data. 

The inclusion criteria, which involved at least one parent participating in the study, were as follows: (a) the appropriate age of the child (3–10 years) in the family, (b) the good health of the participant (absence of any disease that would prevent him/her from attending the regular programme in kindergarten/primary school or employment); and (c) willingness to participate in the research voluntarily and at no cost [18].

### 2.2. Participants and Dataset

Anthropometric data for all family members, along with socioeconomic and demographic characteristics, were collected via a family diary and a questionnaire completed by the parents. Between March 2022 and May 2023, 24 h movement behaviours—including PA, SB, and sleep—were continuously monitored using wrist-worn accelerometers over a 7-day period during a regular kindergarten/school week, excluding multi-day vacations and holidays.

Out of 860 families contacted, 552 provided signed informed consent to participate. Of these, 502 families initiated 7-day 24 h movement behaviour monitoring, and 472 families completed it. Participants were excluded from the final dataset for failing to meet the accelerometer wear-time criteria—defined as at least 16 h per day on a minimum of 3 school days and 1 weekend day (n = 65)—or due to missing anthropometric data, missing gender information, or insufficient data to calculate family SES (n = 26). The final analyzed sample comprised 381 children (49.9% girls) with valid 24 h movement behaviour data and complete anthropometric, socioeconomic, and demographic information (see Table 1).

Parents were instructed by the researchers on how to wear the accelerometer and record 24 h movement behaviour data in a family diary. With respect to school days and weekends, depending on the monitoring period, the family diary was organized in a table format to allow parents to conveniently record time data related to their daily routines for themselves and their child—wake-up time, arrival at kindergarten/school, physical education classes, departure from kindergarten/school, units of organized PA (training sessions, coach/leader-led clubs), bedtime and modes of transport (walking, cycling, car, public transport). Monitoring of 24 h movement behaviour using an accelerometer was initiated at midnight on the day of the researchers’ meeting with parents. During this meeting, parents were instructed to place the accelerometer on themselves and their children before bedtime that same day. At the end of the weekly monitoring, accelerometers and completed family diaries were collected by the researchers.

### 2.3. Measurement of 24 h Movement Behaviour

The 24 h movement behaviours of children and their parents were monitored using accelerometers worn on the wrist of the non-dominant hand, continuously for seven days, excluding periods of bathing and swimming. For the children, we used the ActiGraph wGT3X-BT accelerometers (ActiGraph Corp., Pensacola, FL, USA), and for the parents, we used the ActiGraph GT9X Link accelerometers (ActiGraph LLC, Pensacola, FL, USA). All accelerometers were individually initialized for each family member using dedicated ActiLife software version 6.13.4 (ActiGraph LLC, Pensacola, FL, USA). The devices were configured to capture triaxial acceleration data at a sampling rate of 100 Hz. To avoid confusion, each accelerometer was assigned specifically for a family member.

All accelerometer files were analyzed with R package GGIR version 2.7-1. We utilized previously established cut-off points to categorize the intensity levels of participants’ 24 h movement behaviours. Specifically, SB was defined as acceleration values less than 36 mg; light PA ranged from 36 to 200 mg; moderate PA was identified between 201 and 706 mg; and vigorous PA was classified as values equal to or greater than 707 mg [19,20]. Sleep time, defined as the duration from sleep onset to awakening, was detected using the default settings of a heuristic algorithm that analyses the distribution of changes in the Z-angle [20].

The criteria for the inclusion of accelerometer data in the final dataset required recording at least three school days and one weekend day, with a minimum daily recording time of 16 h. Additionally, accelerometer wear data needed to be available for each 15 min interval of the 24 h cycle [20]. Average daily sleep time, SB, light PA, MVPA, vigorous PA, and total PA were calculated as the weighted arithmetic mean of these activities performed during school and weekend days. The weighted mean was computed using the following formula: Weighted Mean = [(Weekday Mean × 5) + (Weekend Mean × 2)]/7. Total PA is defined as the sum of light PA, moderate PA, and vigorous PA.

### 2.4. Anthropometric Characteristics and Socioeconomic Status of Participants

Informed consent for participation included questions addressed to parents regarding the collection of anthropometric characteristics of the participants. Prior to the start of the 24 h movement behaviour monitoring, parents answered simple questions about basic anthropometric parameters (gender, month and year of birth, and body height/weight to the nearest 0.5 cm/0.1 kg) for each participating family member at home. The measurements of children’s height and weight performed by their parents at home were confirmed as sufficiently valid for calculating body mass index (BMI) [21,22,23] and subsequent identification of overweight and obesity in 4–10-year-old children [21,22].

Six questions about families’ material wealth from the Family Affluence Scale, which were part of the family diary, were used to assess family SES. The original questions used to determine SES were obtained from the international research study named Health Behaviour in School-aged Children [13], then translated, validated, and reused in previous studies including Czech families with children [18,24]. The content of the six questions and their response options were as follows: having one’s own bedroom for each child in the family (0 or 1); number of computers in the household (0, 1, 2 and ≥3); number of cars owned for family use (0, 1 and ≥2); number of foreign holidays taken in the past year (0, 1, 2 and ≥3); ownership of a dishwasher (0 or 1); number of bathrooms in the household (0, 1, 2 and ≥3). The sum of all six questions formed a summary score from which three categories of family SES were calculated as follows: the lowest/highest 20% of the summary score characterized families with “low”/“high” SES, and the range of 21–79% of the summary score identified families with “medium” SES [18,24].

### 2.5. The Ethics

The study was approved by the Ethics Committee of the Faculty of Physical Culture at Palacký University Olomouc on 28 February 2021, ref. no. 25/2021. The approval from the Ethics Committee included the methodological protocol, which contained the conditions of participation in the research as stated in the informed consent for parents and their offspring. The techniques and instruments used were health- and hygiene-compliant and were disinfected before each use. Personal information was anonymized, as were responses on SES and the background of the families’ environment. None of the participants were penalized in case of damage or loss of the accelerometer or interruption and non-completion of the research. Participation in the research was voluntary and free of charge. Each research participant received individualized feedback on the results of the monitoring of his or her own 24 h movement behaviour in the form of graphic sheets with explanatory commentary. The overall results of the study for the kindergarten/school were compiled for the representatives of the participating schools, together with a thank you note and a certificate of participation in the research.

### 2.6. Data Processing and Statistics

**Figure 1:**
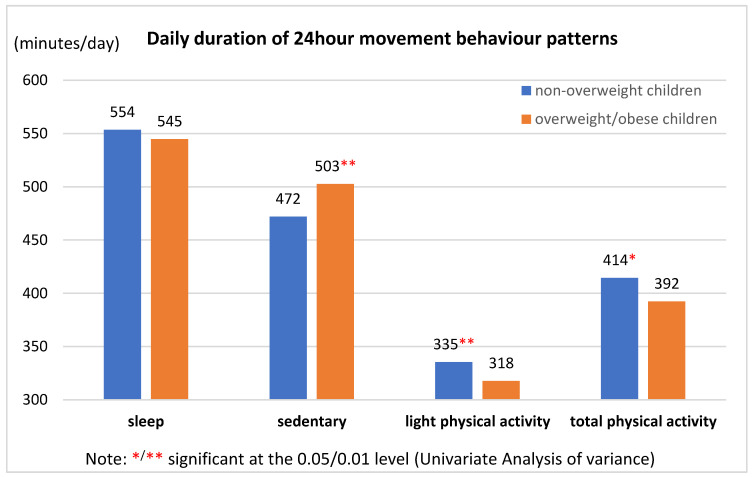
Comparison of 24 h movement behaviour patterns between non-overweight children and children with overweight/obesity.

All data processing and statistical analyses were performed in the software “Statistical Package for the Social Sciences for Windows V.26” (IBM Corp, Armonk, NY, USA). The final sample with valid anthropometric characteristics, PA, SB and sleep data was checked for outliers and obvious errors. The BMI of the children was calculated as the ratio of body weight (kg) to the square of body height (m), and the results were expressed as z-score based on the gender- and age-specific WHO reference data [25]. Children with a BMI z-score > 1 standard deviation (SD) and <2 SDs were classified as overweight, while those with a BMI z-score > 2 SDs were classified as obese [25,26,27]. Overweight and obesity in parents were represented by a BMI in the range of 25–29.9 kg/m^2^ or ≥30 kg/m^2^, respectively [5]. Descriptive characteristics were presented as percentages, arithmetic means, and SDs with respect to the distribution of variables, separately for girls and boys (Table 1), as well as non-overweight (normal weight and underweight) children and children with overweight/obesity (Figure 1). A *t*-test (or Pearson Chi-square test) was used to analyze differences in calendar age (or frequency of overweight/obesity and family SES) between girls and boys. Pearson’s correlation coefficient was used to reveal relationships between 24 h movement behaviour patterns and children’s body weight levels (BMI, BMI z-score, and body weight category). Differences in the daily duration of PA, SB, and sleep (resp., gender, calendar age, SES levels, and the proportion of maternal and paternal obesity) between non-overweight children and children with overweight/obesity were tested by univariate analysis of variance (resp. Pearson Chi-Square test). An alpha α level of 5% was set for all statistical procedures.

## 3. Results

No statistically significant differences were found between girls and boys regarding calendar age (F = 0.144, *p* = 0.497), prevalence of overweight/obesity (χ^2^ = 0.067, *p* = 0.897), or family SES (χ^2^ = 1.977, *p* = 0.372); therefore, subsequent detection of similarities/differences between non-overweight children and children with overweight/obesity can be performed cumulatively for both girls and boys, as well as across different SES categories of families. Regarding the relationship between certain characteristics of children’s body weight levels (BMI, BMI z-score, and body weight category) and 24 h movement behaviour patterns, significant positive associations were observed in SB (r_P_ = 0.112–0.168), while significant negative associations were found in light PA (r_P_= −0.137 to −0.192) and total PA (for BMI and body weight category) (Table 2). Although the statistically significant associations between children’s body weight levels and 24 h movement behaviour patterns were low, the results showed that higher BMI, BMI z-score, and body weight were related with longer sessions of SB. Conversely, lower BMI and body weight in children were linked with longer duration of light and total PA.

Along with the findings on the significant associations between children’s body weight levels and their SB or light/total PA, differences in the daily duration of SB and PA were observed between non-overweight children and children with overweight/obesity (Figure 1). Non-overweight children exhibited 22 more minutes of total PA per day and 31 fewer minutes of SB per day than over-weight/obese children (Figure 1). Differences in daily sleep duration between non-overweight children and children with overweight/obesity were not statistically significant. Mean daily MVPA exceeded 60 min for both non-overweight children and those with overweight/obesity (MVPA_non-overweight_ = 78.93 ± 31.31 min/day; MVPA_overweight/obese_ = 74.85 ± 30.09 min/day). The difference of approximately 4 min per day in MVPA between non-overweight children and children with overweight/obesity was not significant.

In addition to the key finding that non-overweight children had significantly higher PA and lower SB compared to children with overweight/obesity, various other correlates and determinants were tested to explore factors influencing these differences (Table 3). 

Among the potential correlates of childhood excess body weight, the following variables were analyzed: gender and age of the child, parental obesity prevalence, and family SES (Table 3). Except paternal obesity, no significant differences were found between non-overweight children and children with overweight/obesity aged 3–10 years in the other potential correlates of childhood overweight/obesity that were tested (Table 3).

## 4. Discussion

### 4.1. Interpretation of Results in the Context of Previous Studies and Their Implications

The present study sought to uncover differences between non-overweight children and young children with overweight/obesity (if existed) that could be used to design effective prevention and intervention programmes aimed at reducing excess body weight. First, however, it is necessary to compare the prevalence of overweight/obesity in Czech children aged 3–10 years with their peers from other countries that have similar SES or geographical location. Despite the methodological differences in determining overweight/obesity of young children, the prevalence of overweight/obesity in Czech children (20.11% for girls and 19.04% for boys) was similar with that in German 6-year-olds (21.3%) [2], but was higher than that in Polish 5–6-year-olds (11.0%) [1] and Norwegian 5–7-year-olds (13.7%) [7]. Moreover, when compared to data from 27 European countries collected between 2006 and 2016, the prevalence of overweight/obesity in Czech children was lower than the European average (23.2%) [4]. Regarding gender differences, unlike most European countries where girls presented a higher pooled prevalence of overweight/obesity than boys [4], we did not observe any significant gender variations in the prevalence of overweight/obesity among children. Interestingly, the rates of overweight/obesity among Czech girls and boys have remained comparable from 2008 to 2022–2023 [4].

The results of this study showed that Czech children with a relatively low prevalence of excess body weight also demonstrated higher levels of daily PA. Both non-overweight children and children with overweight/obesity exceeded the WHO recommended 60 min of MVPA per day by an average of almost 19 (and 15) minutes, respectively [28,29]. Converting to the WHO recommendations for total daily PA and MVPA time for 3–4 year olds [28] and 5-year-olds and older [29], the 3–10-year-olds we studied were more physically active than their peers from Europe, Asia, Australia, Canada and the United States [30,31]. The explanation for higher levels of PA in Czech children aged 5–8 years, compared to those in their international peers, could be attributed to the mandatory daily kindergarten programme that encourages active engagement such as regular PA (daily walks and active play outdoors and indoors), limiting prolonged SB and its interruptions (i.e., group meal preparation, dressing), and adherence to afternoon sleep [32]. In the first and second year of primary school, the curriculum includes regular physical education lessons (twice a week), active time during school breaks and a free after-school club in the afternoon with the possibility of implementing PA according to the teacher’s choice. Completion of daily classes in kindergarten/school was an inclusion criterion for the child to be part of the final sample in this study.

Comparable PA levels of the children we studied were found in Finnish preschoolers [33], 5–8-year-old children living in the US-affiliated Pacific region [34], and Japanese 3–5-year-old preschoolers [35]. In addition, meeting guidelines for sleep or for both PA and sleep were associated with lower BMI and lower waist circumference in Finnish preschoolers [33], and noncompliance with the 24 h movement behaviour recommendations was related to overweight/obesity in children aged 5–8 years living in the US-affiliated Pacific region [34]. Furthermore, compared to Japanese children aged 3–5 years who adhered to all three 24 h movement guidelines, those who did not comply were more likely to be overweight or obese, even after adjusting for gender and age [35]. In summary, it appears that differences in daily PA and SB with respect to body weight level begin to emerge in early childhood when analyzing 3–10-year-old children who meet the recommendations for 24 h movement behaviour.

Relevant studies that have focused on finding associations between family SES and body weight levels in early childhood with respect to 24 h movement behaviour are rare. A higher risk of overweight or obesity was observed in Polish 5–6-year-old children whose parents had lower education levels and were obese, compared to children of non-obese parents with higher education [1]. Additionally, a study from Germany drew attention to the trend of increasing prevalence of overweight/obesity in 4–6-year-old children, especially from families with a migration background [2]. However, it is not yet possible to say with certainty how the SES of families is associated with the occurrence of overweight/obesity in young children regarding their 24 h movement behaviour patterns.

### 4.2. Strengths and Limitations of the Study and Future Research

The strengths of this study include the representative size (n = 381) of the children aged 3–10 years, which included participants from urban and rural communities in the regions of Bohemia, Moravia and Silesia, and the length of the 24 h monitoring of the participants’ movement behaviour. The validated instrumental monitoring of 24 h movement behaviour of families [18] and the minimal proportion of the cohort of children (n = 26) without data on gender, somatic characteristics or SES of their families are also considered strengths of the study. An undeniable advantage was conducting a pilot study [18], which greatly helped to fine-tune the research design and execution, ensuring it was as efficient as possible for all study participants. Another strength of the study is the planned repeated monitoring of 24 h movement behaviour of participating families after 3 years (March 2025 to May 2026). The results will highlight trends in sleep, PA, SB, and body weight levels, providing further insight into the possible causes of changes in the variables of interest.

One of the limitations of the study is the fact that participation was voluntary, which may have led to a self-selected sample of participants in the research. Therefore, it is reasonable to assume that the participating families may represent a more physically active spectrum of the population with a motivation for a healthier lifestyle. Also, reactivity, i.e., change in routine behaviour due to awareness of being a research participant, may have played a role in monitoring 24 h movement behaviour. We attempted to eliminate the aforementioned limitations by emphasizing the “non-competitive” nature of the research. We did not disclose any values of the 24 h movement behaviour recommendations to parents or children prior to monitoring, emphasizing the importance of capturing of the family’s normal lifestyle. Furthermore, we lack data regarding family structure or siblings, which could be important determinants of childhood overweight or obesity. Another limitation of the research is its cross-sectional design, which does not allow for interpretation of the results on the relationship between 24 h exercise behaviour of parents and their children causally. A potential source of inaccuracy in the anthropometric measurements of the children may have arisen from parents measuring them at home. Even though parental measurement of their children’s height and weight may deviate from objective laboratory measurements at the individual level, it has been considered sufficiently accurate in larger studies for categorizing 3–10-year-old children into normal weight, overweight, and obese groups [22,36]. While parent-reported anthropometric data can be subject to reporting bias, previous research involving Czech families has demonstrated high agreement between parent-reported and objectively measured body weight and height in young children [23]. This suggests that parental reports can serve as a simple and cost-effective tool in large-scale studies without significantly compromising data accuracy. In addition, the wide age range of the children measured may be considered a limitation of the study, but it is required that subsequent longitudinal research follows up on this measurement.

### 4.3. Innovative Aspects of This Study

1. A weeklong objective measurement of 24 h movement behaviours:

An innovative feature of our study was the use of objective continuous monitoring of all components of 24 h movement behaviours over seven consecutive days using wrist-worn accelerometers. This comprehensive approach allowed for a detailed and accurate assessment of children’s movement patterns across an entire week, including both weekdays and weekends.

2. Focusing on young children aged 3 to 10 years:

Our research uniquely focused on young children aged 3 to 10 years, encompassing both preschool and early primary school ages. This age range is critical for establishing healthy movement behaviours and preventing obesity, yet it is understudied in the context of comprehensive 24 h movement monitoring. 

### 4.4. Practical Implications

Our study’s findings may have important implications for future research and intervention strategies targeting childhood overweight and obesity. While we did not find significant differences between non-overweight children and children with overweight/obesity in sleep duration or MVPA, we observed significant differences in SB, LPA, and total PA. These results suggest that interventions aiming to reduce sedentary time and increase LPA throughout the day could potentially be more effective from early childhood. Moreover, promoting changes in SB and LPA may be more feasible and manageable than increasing MVPA in young children, considering their developmental stage and natural activity patterns. However, given the cross-sectional nature of our study and the limited sample size, these findings should be interpreted with caution. Future research involving larger cohorts and longitudinal designs is needed to confirm these results and to explore how modifying SB and LPA influences weight outcomes over time among young children.

## 5. Conclusions

Differences in 24 h movement behaviours, including SB and PA, are observed between non-overweight and overweight/obese Czech children, but not in sleep duration. Non-overweight children engaged in significantly higher levels of PA and lower levels of SB compared to their counterparts with overweight/obesity, independent of gender, age, and family SES. Specifically, they had averages of 22 more minutes of PA and 31 fewer minutes of SB per day. These findings highlight an association between daily movement behaviours and weight status in young children. Given that even modest differences in PA and SB are associated with weight status, interventions aiming to promote PA and reduce SB could be beneficial across diverse demographic groups. However, due to the cross-sectional design of this study, causality cannot be established. Further longitudinal research is needed to explore the potential impact of modifying PA and SB on weight outcomes in this population.

## Figures and Tables

**Table 1:** Basic somatic and family characteristics of the participating children.

Characteristics	Girls (n = 190)	Boys (n = 191)
Age (months)	77.14 ± 20.29	78.52 ± 19.49
Height (cm)	120.13 ± 12.88	121.31 ± 12.23
Weight (kg)	23.43 ± 7.11	23.49 ± 6.10
Body Mass Index (kg·m^−^²)	15.96 ± 2.65	15.76 ± 2.20
Overweight (%)	12.7 (n = 24)	14.8 (n = 28)
Obese (%)	7.4 (n = 14)	4.2 (n = 8)
Socioeconomic Status (%)		
Low	14.8	11.1
Middle	66.1	72.8
High	19.1	16.1
Valid Days of Accelerometer Wear	5.28 ± 0.67	5.27 ± 0.72
Maternal Obesity (%)	8.3	11.4
Paternal Obesity (%)	11.2	16.8

Note: Values are presented as mean ± standard deviation or percentage.

**Table 2:** Associations (r_p_) between 24 h movement behaviour patterns and children’s body weight levels.

	Sleep	SB	Light PA	MVPA	Total PA
BMI	−0.050	0.160 **	−0.192 **	−0.008	−0.145 **
BMI z-score	−0.052	0.112 *	−0.137 **	0.021	−0.092
Body weight category ^#^	−0.082	0.168 **	−0.155 **	−0.048	−0.136 **

Note: r_p_—Pearson’s correlation coefficient; BMI—body mass index (kg·m^−2^); ^#^—a category of 0 = underweight, 1 = normal weight, 2 = overweight, and 3 = obese; SB—sedentary behaviour; PA—physical activity; MVPA—moderate to vigorous PA; * significant at the 0.05 level; ** significant at the 0.01 level.

**Table 3:** Comparison of somatic parameters and socioeconomic status between non-overweight and overweight/obese children.

Children’s Body Weight Level	Gender of the Child		Calendar Age of Child		SES of Families		Maternal Obesity		Paternal Obesity	
	χ^2^	*p*	χ^2^	*p*	χ^2^	*p*	χ^2^	*p*	χ^2^	*p*
Non-overweight Overweight/obese	0.67	0.80	0.50	0.48	3.78	0.15	1.40	0.24	8.28	0.04

Note: SES—socioeconomic status of the children’s families; χ^2^—Pearson Chi-square test; *p*—level of statistical significance.

## Data Availability

The datasets presented in this article are not readily available because this study is part of a longitudinal research project. The baseline phase data are subject to privacy and ethical restrictions as participants in this study have signed consent forms stipulating that individual data will not be made publicly available until five years after the completion of the follow-up phase.
